# Analytical solutions of PDEs by unique polynomials for peristaltic flow of heated Rabinowitsch fluid through an elliptic duct

**DOI:** 10.1038/s41598-022-17044-y

**Published:** 2022-07-28

**Authors:** Salman Akhtar, Muhammad Hasnain Shahzad, Sohail Nadeem, Aziz Ullah Awan, Shahah Almutairi, Hassan Ali Ghazwani, Mohamed Mahmoud Sayed

**Affiliations:** 1 Department of Mathematics, Quaid-I-Azam University, Islamabad, 4532044000 Pakistan; 2 Department of Mathematics, University of the Punjab, Lahore, Pakistan; 3 Mathematics Department, Faculty of Sciences, Northern Border University, Arar, 1321 Saudi Arabia; 4 Department of Mechanical Engineering, Faculty of Engineering, Jazan University, P.O box 45124, Jazan, Kingdom of Saudi Arabia; 5 Architectural Engineering Department, Faculty of Engineering and Technology, Future University in Egypt, New Cairo, 11835 Egypt

**Keywords:** Mathematics and computing, Applied mathematics, Computational science

## Abstract

In this research, we have considered the convective heat transfer analysis on peristaltic flow of Rabinowitsch fluid through an elliptical cross section duct. The Pseudoplastic and Dilatant characteristics of non-Newtonian fluid flow are analyzed in detail. The Rabinowitsch fluid model shows Pseudoplastic fluid nature for $\sigma > 0$ and Dilatant fluid behaviour for $\sigma < 0.$ The governing equations are transformed to dimensionless form after substituting pertinent parameters and by applying the long wavelength approximation. The non-dimensional momentum and energy equations are solved analytically to obtain the exact velocity and exact temperature solutions of the flow. A novel polynomial of order six having ten constants is introduced first time in this study to solve the energy equation exactly for Rabinowitsch fluid flow through an elliptic domain. The analytically acquired solutions are studied graphically for the effective analysis of the flow. The flow is found to diminish quickly in the surrounding conduit boundary for Dilatant fluid as compared to the Pseudoplastic fluid. The temperature depicted the opposite nature for Pseudoplastic and Dilatant fluids. The flow is examined to plot the streamlines for both Pseudoplastic and Dilatant fluids by rising the flow rate.

## Introduction

The process in which fluid flows across a conduit by the sinusoidal fluctuating boundaries is known as Peristalsis. The fluid propels by the means of deformation of the walls parallel to the axis of channel in peristaltic flow. The wide applications of peristaltic flow in the industrial area, physiological and engineering fields like corrosive fluid transport, blood pumps in heart lungs machines, blood flow in small vessels makes it much important. Due to these many applications numerous researchers has been studying the peristaltic flow via various conduits. Bohm and Friedrich studied the flow of viscoelastic fluids under peristaltic wall deformation of channel by considering an assumption of small Reynold number^1^. Siddiqui and Schwarz provided analytical study of the Second-grade fluid flow through axisymmetric tubes with sinusoidally vibrating walls^2^. Eytan et al. described the peristaltic flow via narrowed conduit under the application of embryo transport within the uterine aperture^3^. Tsiklauri and Beresnev had considered the flow of Maxwell fluid across a circular channel with peristaltic movement of the boundary^4^. Rao and Mishra explained the asymmetric channel flow in addition to viscous effects and without neglecting the curvature effects^5^. Reddy et al. analyzed fluid flow due to fluctuating walls of rectangular shape channel by considering long wavelength approximation^6^. The fractional model of Maxwell fluid is utilized to study the visco-elastic fluid flow inside a sinusoidal wavy conduit by Tripathi et al.^7^.

Ali et al. provided the analysis of temperature distribution within a curved wavy conduit^8^. Akbar et al.^9^ examined the entropy generation and MHD influences on peristaltic flow. Maraj and Nadeem^10^ interpreted the curved channel peristaltic flow of non-Newtonian Rabinowitsch fluid. Hayat et al.^11^ explained the entropy interpretation of peristaltic flow of nanofluid in a rotating frame. Rashid et al.^12^ explained the Williamson fluid flow via curved conduit under the influence of magnetic and peristaltic effects. Saleem et al.^13^ studied the Casson fluid to highlight the non-Newtonian nature of fluid via duct with elliptical cross section and fluid flows due to peristaltic effects. McCash et al.^14^ provided the analysis of Newtonian fluid flow through elliptical duct affected by cilia. Asha and Beleri^15^ highlighted the nanofluid effects on non-Newtonian Carreau model for an axially symmetric inclined conduit by contemplating the Joule heating effect. Riaz et al.^16^ gave the analysis of solid particles influence on the peristaltically flowing Jeffery fluid across eccentric annuli. Vaidya et al.^17,18^ studied the influence of variable liquid and rheological characteristics on peristaltic flow of Rabinowitsch fluid across an inclined channel. The variables properties and application of peristaltic flow of non-Newtonian Rabinowitsch fluid under various effects is discussed by many researchers^19–22^.

The heat transfer effects on fluid flowing through different channels are studied by many researchers. The peristaltic flow of heated magnetohydrodynamic fluid under partial slip influence is analyzed by Nadeem and Akram^23^. Akbar and Butt^24^ provided the analytical study of nanofluid flow in curved tube with vibrating walls. Bibi and Xu^25^ presented the heat transfer in a nanofluid flow through a horizontal conduit with MHD effects. Raza et al.^26^ gave the study of water-based nanofluid for various shaped nanoparticles in an asymmetric conduit having permeable boundary under magnetic effects. The influence of heat flux and endoscope on non-Newtonian Jeffery fluid movement across concentric symmetric tubes due to peristaltic motion of walls is discussed by Abd-Alla and Abo-Dahab^27^. Abbasi et al.^28^ examined the nanofluids flow under the peristaltic and electroosmotic effects via asymmetric narrow channel. Li et al.^29^ studied the peristaltic movement of non-Newtonian nanofluid by utilizing Jeffery fluid model under the hall effects and viscous dissipation. Some of the recent literature work that highlights the peristaltic flow, pseudo plastic flow characteristics, MHD impacts and chemical reactions on a channel flow problem are given as^30–33^.

In this research, we have studied the heated Rabinowitsch fluid flow across a duct of elliptical cross section. The published literature and researchers clearly highlight that there is no research on Peristaltic flow of heated Rabinowitsch fluid inside an elliptic duct. The non-Newtonian fluid flow is studied through a channel with sinusoidally fluctuating boundary walls. The velocity and heat transfer effects on fluid flow are analyzed. A unique polynomial is involved to obtain the solution of temperature. The dilatant and pseudoplastic characteristics of the Rabinowitsch fluid are examined.

## Mathematical formulation

**Figure 1:**
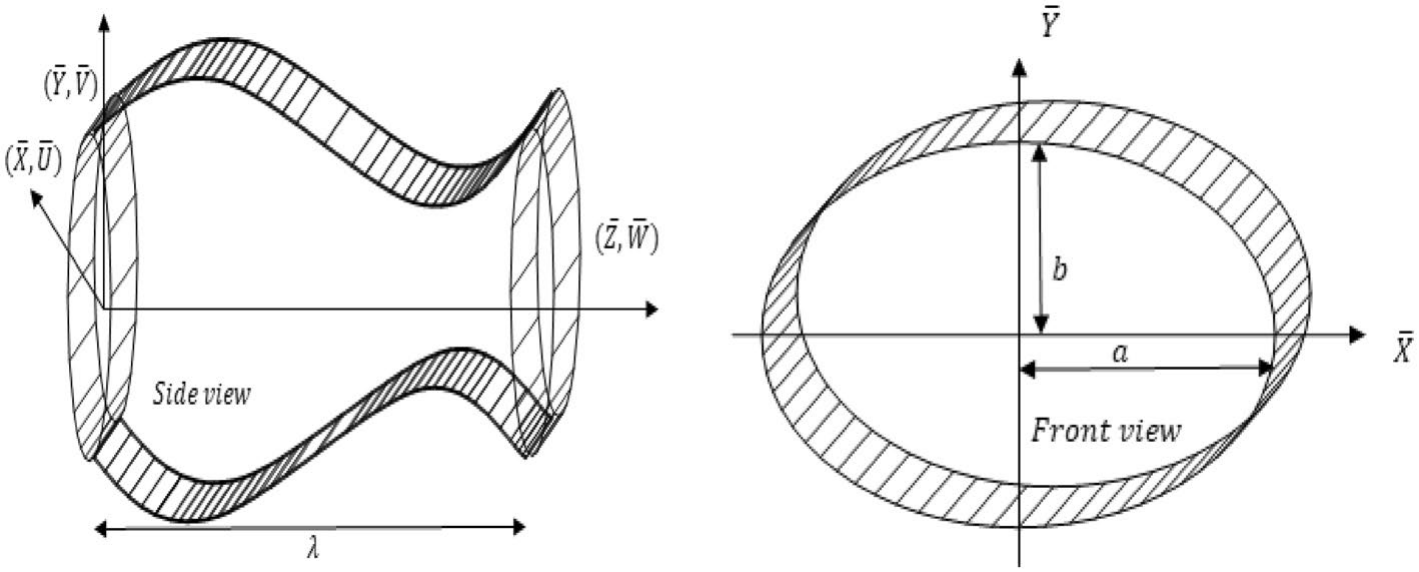
Geometry of Elliptic Duct.

Consider the flow of a heated Rabinowitsch fluid through a duct having elliptical cross section. The elliptic shape conduit is studied with sinusoidally fluctuating walls. The mathematical study in completed by utilizing cartesian coordinates $(X, Y,Z)$. The sinusoidally deformed walls are taken as^34^ and the geometrical model is given by Fig. 1

1-a $${\text{a}}\left( {{\text{Z}},{\text{t}}} \right) = a_{0} + d\sin \left( {{ }\frac{{2{\uppi }}}{\lambda }\left( {{\text{Z}} - {\text{ct}}} \right)} \right),$$

1-b $${\text{b}}\left( {{\text{Z}},{\text{t}}} \right) = b_{0} + d\sin \left( {{ }\frac{{2{\uppi }}}{\lambda }\left( {{\text{Z}} - {\text{ct}}} \right)} \right),$$

The governing equations of incompressible fluids are

2 $$\frac{\partial U}{{\partial X}} + \frac{\partial V}{{\partial Y}} + \frac{\partial W}{{\partial Z}} = 0,$$

3 $$\rho \left( {\frac{\partial U}{{\partial t}} + U\frac{\partial U}{{\partial X}} + V\frac{\partial U}{{\partial Y}} + W\frac{\partial U}{{\partial Z}}} \right) = - \frac{\partial P}{{\partial X}} + \frac{{\partial \tau_{XX} }}{\partial X} + \frac{{\partial \tau_{XY} }}{\partial Y} + \frac{{\partial \tau_{XZ} }}{\partial Z},$$

4 $$\rho \left( {\frac{\partial V}{{\partial t}} + U\frac{\partial V}{{\partial X}} + V\frac{\partial V}{{\partial Y}} + W\frac{\partial V}{{\partial Z}}} \right) = - \frac{\partial P}{{\partial Y}} + \frac{{\partial \tau_{YX} }}{\partial X} + \frac{{\partial \tau_{YY} }}{\partial Y} + \frac{{\partial \tau_{YZ} }}{\partial Z},$$

5 $$\rho \left( {\frac{\partial W}{{\partial t}} + U\frac{\partial W}{{\partial X}} + V\frac{\partial W}{{\partial Y}} + W\frac{\partial W}{{\partial Z}}} \right) = - \frac{\partial P}{{\partial Z}} + \frac{{\partial \tau_{ZX} }}{\partial X} + \frac{{\partial \tau_{ZY} }}{\partial Y} + \frac{{\partial \tau_{ZZ} }}{\partial Z},$$

6 $$\begin{aligned} {\uprho }c_{p} { }\left( \frac{\partial T}{{\partial t}}+{U\frac{{\partial {\text{T}}}}{{\partial {\text{X}}}} + {\text{V}}\frac{{\partial {\text{T}}}}{{\partial {\text{Y}}}} + {\text{W}}\frac{{\partial {\text{T}}}}{{\partial {\text{Z}}}}} \right) = & k\left( {\frac{{\partial^{2} T}}{{\partial X^{2} }} + \frac{{\partial^{2} T}}{{\partial Y^{2} }} + \frac{{\partial^{2} T}}{{\partial \overline{z}^{2} }}} \right) + \left( {\tau_{XX} \frac{\partial U}{{\partial {\text{X}}}} + \tau_{XY} \frac{\partial U}{{\partial {\text{Y}}}} + \tau_{XZ} \frac{\partial U}{{\partial {\text{Z}}}}} \right) \\ & + \left( {\tau_{YX} \frac{\partial V}{{\partial {\text{X}}}} + \tau_{YY} \frac{\partial V}{{\partial {\text{Y}}}} + \tau_{YZ} \frac{\partial V}{{\partial {\text{Z}}}}} \right) + \left( {\tau_{ZX} \frac{\partial W}{{\partial {\text{X}}}} + \tau_{ZY} \frac{\partial W}{{\partial {\text{Y}}}} + \tau_{ZZ} \frac{\partial W}{{\partial {\text{Z}}}}} \right), \\ \end{aligned}$$

The boundary conditions corresponding to above equations are

7 $$W = 0, T = 0 for \frac{{X^{2} }}{{a^{2} }} + \frac{{Y^{2} }}{{b^{2} }} = 1$$

The Rabinowitsch fluid stress tensor is provided as^17^

8 $$\tau_{YZ} + \sigma \tau_{YZ}^{3} = \mu \frac{{\partial {\text{W}}}}{{\partial {\text{Y}}}} ,$$

where, $\sigma$ is coefficient of pseudoplasticity. The Rabinowitsch fluid shows Pseudoplastic, Newtonian, Dilatant behaviour for $\sigma > 0, \sigma = 0, \sigma < 0$ respectively.

The transformations relate the fixed (unsteady) and wave (steady) frames are given following

9 $$\begin{gathered} U = u, V = v, W - c = w , \hfill \\ P = p, X = x, Y = y, z = Z - ct, \hfill \\ \end{gathered}$$

The dimensionless variables to transform the equations into non-dimensional form

10 $$\begin{gathered} \overline{x} = \frac{x}{{D_{h} }} , \overline{y} = \frac{y}{{D_{h} }} , \overline{z} = \frac{z}{\lambda } , \overline{t} = \frac{ct}{\lambda } , \hfill \\ \overline{a} = \frac{a}{{D_{h} }} , \delta = \frac{{b_{0} }}{{a_{0} }} , \overline{p} = \frac{{D_{h}^{2} p}}{\mu \lambda c} , \phi = \frac{d}{{b_{0} }} , \hfill \\ \overline{b} = \frac{b}{{D_{h} }} , \overline{u} = \frac{\lambda u}{{D_{h} c}} , \overline{v} = \frac{\lambda v}{{D_{h} c}} , \overline{w} = \frac{w}{c} , \hfill \\ {\overline{\sigma }} = \frac{{\sigma c^{2} \mu^{2} }}{{D_{h}^{2} }}, \theta = \frac{{T - T_{w} }}{{T_{b} - T_{w} }} , \overline{s}_{ij} = \frac{{D_{h} }}{{c\mu_{f} }}s_{ij} , \hfill \\ B_{r} = \frac{{\mu u_{0}^{2} }}{{k\left( {T_{b} - T_{w} } \right)}} , \hfill \\ \end{gathered}$$

where, $D_{h}$ is the hydraulic diameter of the ellipse and

11 $$D_{h} = \frac{{\pi b_{0} }}{E\left( e \right)} .$$

*e* is known as the eccentricity of the ellipse such that $0 < e < 1$ and $e = \sqrt {1 - \delta^{2} }$

12 $$E\left( e \right) = \mathop \int \limits_{0}^{{\frac{\pi }{2}}} \sqrt {1 - e^{2} Sin^{2} \left( \alpha \right)} d\alpha .$$

By applying long wavelength approximation together with the transformation given in (9) and non-dimensional variables by ignoring dash notation provided in (10), Eqs. (2)-(7) takes the form

13 $$\frac{\partial p}{{\partial x}} = 0,$$

14 $$\frac{\partial p}{{\partial y}} = 0,$$

15 $$\frac{\partial p}{{\partial z}} + \frac{{\partial \tau_{xz} }}{\partial x} + \frac{{\partial \tau_{yz} }}{\partial y} = 0,$$

16 $$\frac{{\partial^{2} \theta }}{{\partial x^{2} }} + \frac{{\partial^{2} \theta }}{{\partial y^{2} }} + B_{r} \left[ {\tau_{xz} \frac{ \partial w}{{\partial {\text{x}}}} + \tau_{yz} \frac{ \partial w}{{\partial {\text{y}}}}} \right] = 0,$$

The corresponding dimensionless boundary conditions are

17 $$w = 0 , \theta = 0 \frac{{x^{2} }}{{a^{2} }} + \frac{{y^{2} }}{{b^{2} }} = 1,$$

The expression (1-a) and (1-b) take the form

18-a $$a = \frac{E\left( e \right)}{\pi }\left( {\frac{1}{\delta } + \phi {\text{Sin}}\left( {2\pi z} \right)} \right),$$

18-b $$b = \frac{E\left( e \right)}{\pi }\left( {1 + \phi \sin \left( {2\pi z} \right)} \right),$$

The dimensionless form of extra stress tensor for Rabinowitsch fluid is

19 $$\tau_{yz} + \sigma .\left( {\tau_{yz} } \right)^{3} = \frac{\partial w}{{\partial y}} ,$$

## Exact solution

### Axial velocity

The following Eq. (20) represents the solution of Eq. (15) with the boundary conditions $\tau_{xz} = 0$ at $x = 0$ and $\tau_{yz} = 0$ at $y = 0$.

20 $$\tau_{xz} = \frac{\partial p}{{\partial z}}\frac{x}{2} {\text{and}} \tau_{yz} = \frac{\partial p}{{\partial z}}\frac{y}{2},$$

The velocity solution is obtained by using Eq. (20) and direct integration of Eq. (19) as

21 $$w\left( {x,y} \right) = \frac{{b^{2} }}{4}\frac{\partial p}{{\partial z}}\left( {\frac{{x^{2} }}{{a^{2} }} + \frac{{y^{2} }}{{b^{2} }} - 1} \right) + \frac{{\sigma b^{4} }}{32}\left( {\frac{{x^{2} }}{{a^{2} }} + \frac{{y^{2} }}{{b^{2} }} - 1} \right)\left( { - \frac{{x^{2} }}{{a^{2} }} + \frac{{y^{2} }}{{b^{2} }} + 1} \right).$$

The volumetric flow rate is attained by integrating the Eq. (21) over elliptic domain

22 $$q\left( z \right) = - \frac{{ab^{3} \pi \frac{\partial p}{{\partial z}}}}{64}\left( {8 + b^{2} \left( {\frac{\partial p}{{\partial z}}} \right)^{2} \sigma } \right)$$

The mathematical expression for pressure gradient is attained by utilizing the equation $q\left( z \right) = Q - \mathop \smallint \limits_{0}^{1} abdz$,

23 $$\begin{aligned} \frac{dp}{{dz}} = & \frac{1}{{3ab^{5} \sigma \left( {18\pi a^{2} b^{10} \left( {L - Q} \right)\sigma^{2} + \pi \sqrt {6a^{4} b^{20} \sigma^{3} \left( {a^{2} b^{4} \pi^{2} + 54\left( {L - Q} \right)^{2} \sigma } \right)} } \right)^{\frac{1}{3}} }} \\ \times \left[ {2a^{2} b^{8} \left( {6\pi } \right)^{2/3} \sigma - 2\left\{ {6^{1/3} \left( {18a^{2} b^{10} \left( {L - Q} \right)\sigma^{2} + \sqrt {6a^{4} b^{20} \sigma^{3} \left( {a^{2} b^{4} \pi^{2} + 54\left( {L - Q} \right)^{2} \sigma } \right)} } \right)^{\frac{2}{3}} } \right\}} \right], \\ \end{aligned}$$

where, $$L = \mathop \smallint \limits_{0}^{1} ab dz$$.

The expression for pressure rise is obtained as

$$\vartriangle p = \mathop \smallint \limits_{0}^{1} \frac{dp}{{dz}},$$

### Temperature profile

The solution of temperature is attained by using the method provided in^35^. It is obtained by utilizing the following polynomial

24 $${\uptheta }\left( {{\text{x}},{\text{y}}} \right) = c_{1} x^{6} + c_{2} y^{6} + c_{3} x^{4} + c_{4} y^{4} + c_{5} x^{2} + c_{6} y^{2} + c_{7} x^{4} y^{4} + c_{8} x^{2} y^{2} + c_{9} \left( {x^{4} y^{2} - x^{2} y^{4} } \right) + c_{10} ,$$

By setting the polynomial in Eq. (16) and taking coefficient of $x^{4}$, $x^{2}$, $x^{0}$, $y^{4}$, $y^{2}$, $y^{0}$ equal to zero, we have

25 $$- \frac{{b^{4} B_{r} \sigma }}{{16a^{4} }}\left( {\frac{dp}{{dz}}} \right)^{4} + 30c_{1} + 12y^{2} c_{7} + 2c_{9} = 0,$$

26 $$\frac{1}{16}B_{r} \left( {\frac{dp}{{dz}}} \right)^{4} \sigma + 30c_{2} + 12x^{2} c_{7} - 2c_{9} = 0,$$

27 $$\frac{{b^{2} B_{r} }}{{4a^{2} }}\left( {\frac{dp}{{dz}}} \right)^{2} + \frac{{b^{4} B_{r} \sigma }}{{16a^{2} }}\left( {\frac{dp}{{dz}}} \right)^{4} + 12c_{3} + 12y^{4} c_{7} + 2c_{8} = 0,$$

28 $$\frac{{B_{r} }}{4}\left( {\frac{dp}{{dz}}} \right)^{2} + 12c_{4} + 12x^{4} c_{7} + 2c_{8} = 0,$$

29 $$c_{5} + c_{6} = 0,$$

Moreover, using boundary condition and comparing the coefficients of $x^{8}$, $x^{6}$, $x^{4}$, $x^{2}$, $x^{0}$, we obtain

30 $$\frac{{b^{4} c_{7} }}{{a^{4} }} = 0,$$

31 $$c_{1} - \frac{{b^{6} c_{2} }}{{a^{6} }} - \frac{{2b^{4} c_{7} }}{{a^{2} }} - \frac{{b^{2} c_{9} }}{{a^{2} }} - \frac{{b^{4} c_{9} }}{{a^{4} }} = 0,$$

32 $$\frac{{3b^{6} c_{2} }}{{a^{4} }} + c_{3} + \frac{{b^{4} c_{4} }}{{a^{4} }} + b^{4} c_{7} - \frac{{b^{2} c_{8} }}{{a^{2} }} + b^{2} c_{9} + \frac{{2b^{4} c_{9} }}{{a^{2} }} = 0,$$

33 $$- \frac{{3b^{6} c_{2} }}{{a^{2} }} - \frac{{2b^{4} c_{4} }}{{a^{2} }} + c_{5} - \frac{{b^{2} c_{6} }}{{a^{2} }} + b^{2} c_{8} - b^{4} c_{9} = 0,$$

34 $$b^{6} c_{2} + b^{4} c_{4} + b^{2} c_{6} + c_{10} = 0,$$

The solution of temperature is acquired by solving the Eqs. (25)–(34) simultaneously and putting the value of constants in Eq. (24) as

35 $$\begin{aligned} \theta \left( {x,y} \right) = & \frac{{ - a^{2} b^{2} }}{{960a^{4} \left( {a^{2} + b^{2} } \right)\left( {a^{4} + 6a^{2} b^{2} + b^{4} } \right)\left( {a^{4} + 14a^{2} b^{2} + b^{4} } \right)}} \\ & \times \left[ {B_{r} \left( {\frac{{dp}}{{dz}}} \right)^{2} \left( {\frac{{x^{2} }}{{a^{2} }} + \frac{{y^{2} }}{{b^{2} }} - 1} \right)\left( {\left( {20a^{2} \left( {a^{4} + 14a^{2} b^{2} + b^{4} } \right)\left( {b^{6} x^{2} } \right.} \right.} \right.} \right. \\ & \left. { + a^{6} \left( {b^{2} + y^{2} } \right) + a^{2} b^{4} \left( {b^{2} + 6x^{2} + 5y^{2} } \right) + a^{4} b^{2} \left( {10b^{2} + 5x^{2} + 6y^{2} } \right)} \right) \\ & + \left( {\frac{{dp}}{{dz}}} \right)^{2} \left( {b^{4} \left( {a^{4} \left( {2a^{8} + 61a^{6} b^{2} + 355a^{4} b^{4} + 59a^{2} b^{6} + 3b^{8} } \right)} \right.} \right. \\ & \left. { + a^{2} \left( {a^{2} + b^{2} } \right)\left( {26a^{6} + 231a^{4} b^{2} + 60a^{2} b^{4} + 3b^{6} } \right)x^{2} - 2\left( {a^{2} + b^{2} } \right)\left( {14a^{2} + b^{2} } \right)\left( {a^{4} + 6a^{2} b^{2} + b^{4} } \right)x^{4} } \right) \\ &\left. { + a^{2} b^{2} \left( {a^{2} + b^{2} } \right)(2a^{8} + 35a^{6} b^{2} + 124a^{4} b^{4} - a^{2} b^{6} - 2\left( {a^{2} - b^{2} } \right)\left( {a^{4} + 6a^{2} b^{2} + b^{4} } \right)x^{2} } \right)y^{2} \\ & \left. {\left. {\left. {\left. { + 2a^{4} \left( {a^{2} + b^{2} } \right)\left( {a^{2} + 14b^{2} } \right)\left( {a^{4} + 6a^{2} b^{2} + b^{4} } \right)y^{4} } \right)\sigma } \right)} \right)} \right] \\ \end{aligned}$$

## Results and discussion

**Figure 2:**
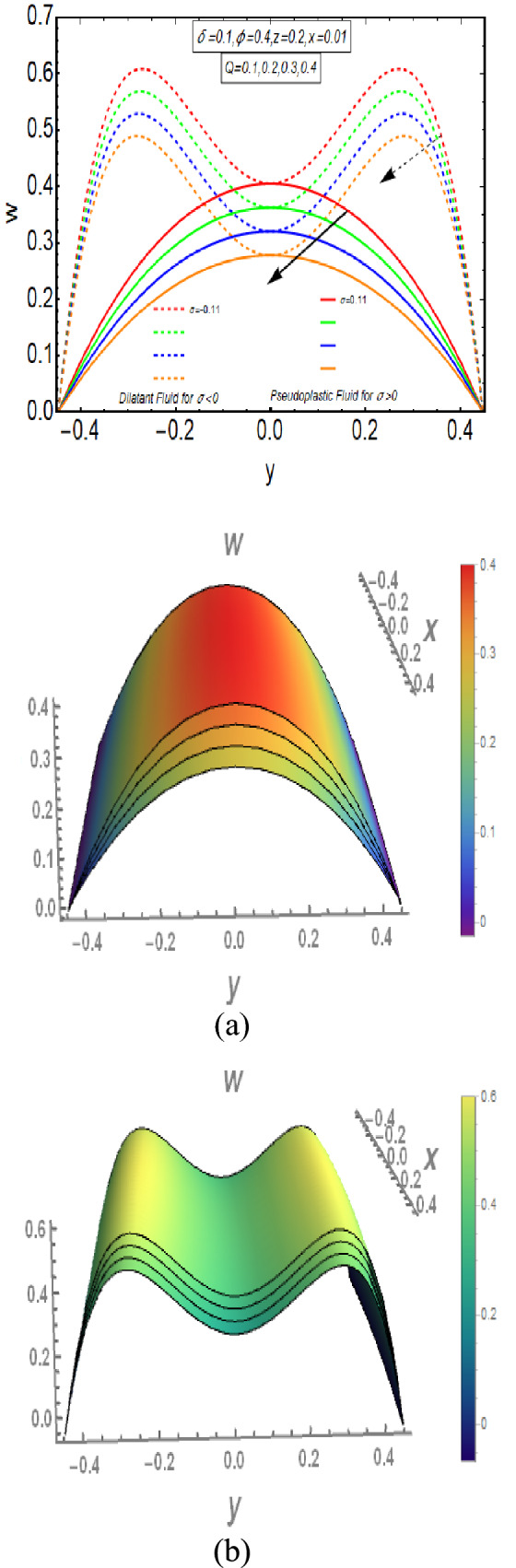
Velocity Profile for $Q$ with $\sigma > 0$ and $\sigma < 0$. (**a**) *σ*  > 0, (**b**) *σ*  < 0.

The graphical analysis of analytically attained results in earlier fragment is presented in this section. This analysis provides an effective study and better understanding of the physical aspects of peristaltic motion of Rabinowitsch fluid flow across elliptic duct. The effect of different physical constraints on the flow velocity, temperature distribution, pressure gradient and pressure rise are discussed. The three-dimensional graphs of velocity and temperature distribution are provided in this graphical examination. Figure 2 has a graphical analysis of the flow velocity for rising values of $Q$. It explains that the velocity of flowing fluid decreases for rapid flow. The velocity of Dilatant fluid has greatest magnitude near the wall of the conduit and diminishes very quickly towards the walls to approach its lowest value. But velocity of Pseudoplastic fluid is maximum at mean of conduit and diminishes gradually towards the walls to get its smallest magnitude. The graph of Dilatant fluid grows fast as compared to the Pseudoplastic fluid. Moreover, flow velocity of Pseudoplastic and Dilatant fluids are same on the axis of channel.

**Figure 3:**
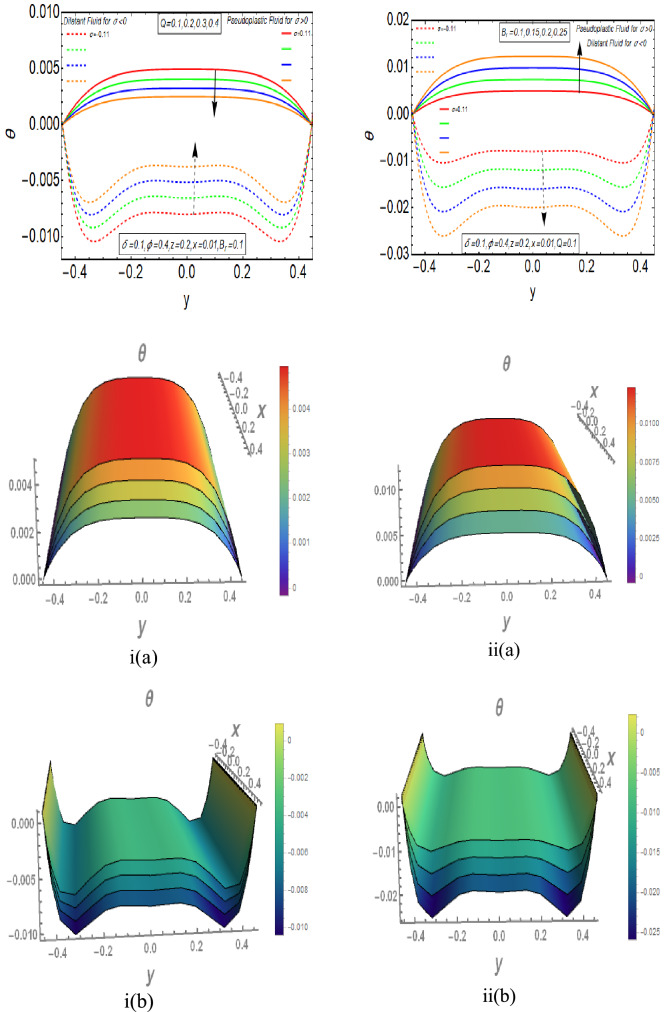
(**i**) Temperature graph for $Q$ with $\sigma > 0$ and $\sigma < 0$, i(**a**) *σ*  > 0, i(**b**) σ < 0. (**ii**)Temperature graph for *Br* with *σ*  > 0 and *σ*  < 0, ii(**a**) *σ*  > 0, ii(**b**) *σ*  < 0.

The Fig. 3i,ii provides the information about the behavior of temperature profile of Rabinowitsch fluid flow. Figure 3i shows the belonging of temperature profile with flow rate $Q$. The magnitude of temperature diminishes by improving the values of flow rate $Q$ for Pseudoplastic behaviour of Rabinowitsch fluid. It remains same near the mid of channel and declines quickly close to the boundary walls. But the Rabinowitsch fluid shows opposite nature for its Dilatant behavior. Figure 3ii depicts the effect of Brinkman number on temperature graph. It delineates that temperature profile reduces for enlarging values of Brinkman number in the case of Dilatant fluid. It rises rapidly in surrounding of deformed wall and remains similar away from it. The temperature of Pseudoplastic fluid enhances by incrementing the values of Brinkman number. The temperature graph of Pseudoplastic and Dilatant fluid shows opposite behavior for increasing magnitude of $B_{r}$.

**Figure 4:**
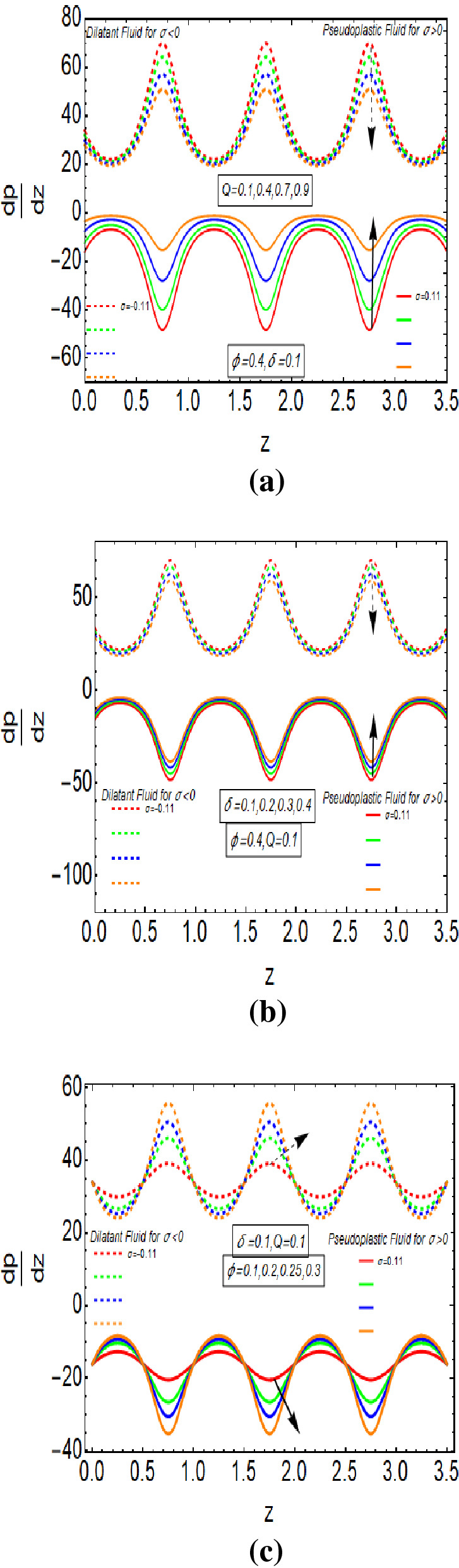
(**a**) $\frac{dp}{{dz}}$ for $Q$ with $\sigma > 0$ and $\sigma < 0$. (**b**) $\frac{{{\text{dp}}}}{{{\text{dz}}}}$ for $\delta$ with $\sigma > 0$ and ${\upsigma } < 0$. (**c**) $\frac{dp}{{dz}}$ for $\varphi$ with $\sigma > 0$ and $\sigma < 0$.

Figure 4a–c reveals the dependence of pressure gradient on the flow rate, aspect ratio and occlusion. Figure 4a explains that pressure gradient graph gets down by increasing the flow rate for Dilatant behavior whereas it gets height for Pseudoplastic behavior of Rabinowitsch fluid. Figure 4b represents that the pressure gradient has larger magnitude by increasing the values of $\delta$ for the Pseudoplastic fluid. But it gives reverse behaviour in the cases of Dilatant fluid. Figure 4c indicates the nature of the pressure gradient graph for growing values of $\varphi$. Its graph is increasing with $0 < z < 0.5$ and it diminishes with $0.5 < z < 1$ for the larger values of $\varphi$ in the case of Pseudoplastic fluid. It shows similar behaviour for other values $z$. But the pressure gradient has opposite nature for Dilatant fluid. The pressure gradient has maximum and minimum values for Dilatant and Pseudoplastic fluid at $z = n + 0.75$, n is whole number, respectively.

**Figure 5:**
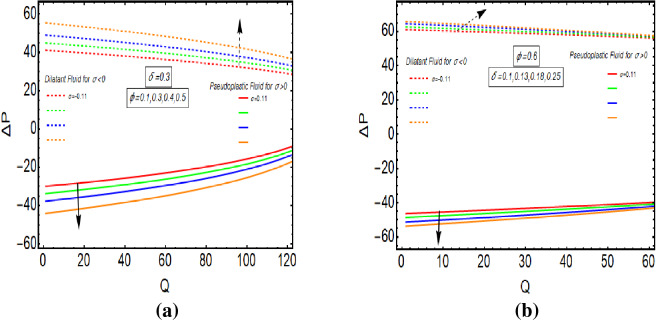
(**a**) Pressure rise for $\varphi$ with $\sigma > 0$ and $\sigma < 0$. (**b**) Pressure rise for *δ* with *σ* > 0 and *σ* < 0.

**Figure 6:**
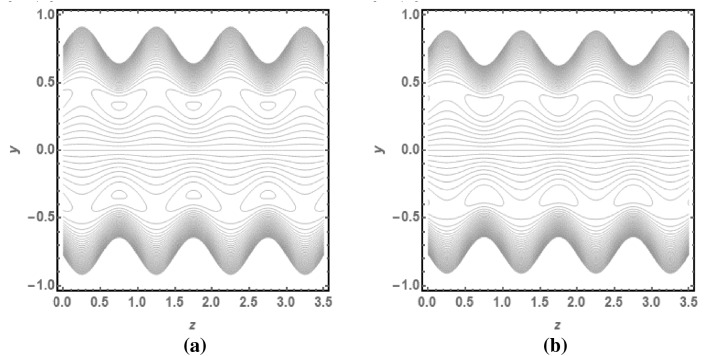
(**a**) Streamline for $Q = 1.4$ and $\sigma > 0$. (**b**) Streamline for $Q = 1.6$ and $\sigma > 0$.

**Figure 7:**
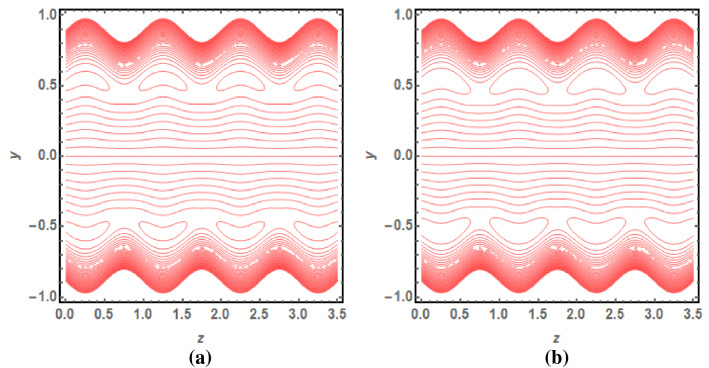
(**a**) Streamline for $Q = 1.4$ and $\sigma < 0$. (**b**) Streamline for $Q = 1.6$ and $\sigma < 0$.

Figure 5a,b provides the graphical representation of pressure rise for $\delta$ and $\varphi$. Figure 5a reveals the enhancing behaviour of pressure rise by incrementing the values of $\delta$ for Dilatant nature. But it has opposite behavior for pseudoplastic nature of fluid. Figure 5b depicts the increasing graph of pressure rise for improving values of $\varphi$. It shows that Dilatant and Pseudoplastic fluids has opposite nature as a function of $\varphi$. The value of $\Delta p$ gets closer to each other for increasing values of $Q$. Figures 6a, 7b provides the streamline analysis of Rabinowitsch fluid through an elliptic duct under the peristaltic motion. Figure 6a,b shows the generation of trappings for various values of flow rate. They clarify that number and size of vortices reduces for higher values of $Q$ in the case of Pseudoplastic fluid flow. Figure 7a,b indicates the existence of trappings in the flow. They show that the rise in flow rate causes to increase in the size of vorticed produces during the flow.

## Conclusions

In this study, we have considered the flow of heated Rabinowitsch fluid across an elliptical cross section duct. The flow is studied with sinusoidally vibrating walls of the conduit. The impact of physical constraints on Pseudoplastic and Dilatant fluids through elliptic duct is analyzed. A novel mathematical technique is introduced to solve the partial differential equation that includes the heat transfer in present analysis. A polynomial of order six with ten constants is introduced first time in such analysis to solve the temperature equation. The major outcomes of our study are following:

- The velocity of flow has same magnitude on the axis of conduit.
- The flow velocity decreases rapidly in neighboring of walls for Dilatant fluid than Pseudoplastic fluid.
- The temperature has opposite behavior for Pseudoplastic and Dilatant fluids.
- Our major task was to solve the complex partial differential equation involved in convection heat transfer and we have provided an exact solution for temperature equation by considering a polynomial of order six with ten constants. Such a polynomial is considered first time in this work to solve the energy equation exactly.
- The pressure gradient shows the periodic behavior.
- The pressure rise nature of Dilatant fluid opposes the behavior of pressure rise of Pseudoplastic fluid.
- The velocity and temperature profiles are parabolic in nature for Pseudoplastic fluid.
- The streamlines analysis unfolds that size of trappings reduces with rise in flow rate for Pseudoplastic fluid but has opposite behavior for Dilatant fluid.

## List of symbols

$$\left( {X,Y,Z} \right)$$: Cartesian coordinates
$$d$$: Wave amplitude
$$D_{h}$$: Hydraulic diameter
$$T_{b}$$: Bulk temperature
$a_{0} ,b_{0}$: Semi axes of ellipse
$$\delta$$: Aspect-ratio
$$\mu$$: Dynamic viscosity
$\sigma$: Coefficient of pseudoplasticity
$$\left( {U,V,W} \right)$$: Velocity components
$$\lambda$$: Wavelength
$$e$$: Eccentricity
$$T_{w}$$: Wall temperature
$c$: Velocity of propagation
$$\phi$$: Occlusion
$$B_{r}$$: Brinkman number
